# The Physiology of Œdema

**Published:** 1892-03-05

**Authors:** 


### THE PRACTITIONER'S MIRROR AND RETROSPECT.

[*The Editor will be glad to receive offers of co-operation and contributions from members of the profession. All letters should be addressed to* THE EDITOR, THE LODGE, PORCHESTER SQUARE, LONDON, W.]

### MEDICINE.

### THE PHYSIOLOGY OF ŒDEMA.

It has been stated that our tissues live in a "running stream of water," and this is literally true, for the fluid portion of the blood which constantly exudes through the capillary walls can only fulfil its function of nourishment if it is as constantly re-absorbed into the veins or lymphatics. Hence the "parenchymatous lymph," that is, the juices bathing the fibres and cells of the body, is always in movement, carrying food to the tissues and removing waste matter from them. Healthy nutrition would therefore appear to be easily understood; the nutriment is in the circulating blood; it exudes as liquor sanguinis through the soft capillary coats, and the surplus, together with the products of tissue metabolism are removed by both veins and lymphatics. The positive *a tergo* pressure in the arteries accounts for the transudation; the low pressure in the veins and capillary lymphatics permits the re-absorption.

The part played by the absorbents, however, is not so clear. Majendie found by actual experiment, that when all the lymphatics of a part were ligatured, no œdema followed, but absorption went on as well as before. Ranvier failed to produce dropsy, even after ligature of the inferior vena cava; but when he also cut the sciatic nerves, and by so doing lowered the blood pressure by vaso-motor paralysis, the legs became œdematous.

From these experiments it would appear that the lymphatics and the veins can mutually replace each other as absorbents, but that dilatation of the arterioles is an important factor.

But disease shows that these mechanical considerations of blood pressure, &c., are inadequate to account for the presence of œdema in certain conditions; and it is probable that the normal return of the effused fluid into the circulation is a more complicated problem than was at first thought.

Does chemical analysis help us? Serous effusions undergo change like other organic fluids, and consequently certain decomposition products are found, such as Leucin, Tyrosin, Xanthin, &c. Pleuritic effusions often contain sugar, and the amount of albumen varies greatly. In œdema of a supposed obstructive origin there is usually very little, whereas in some forms of dropsy there is plenty of both serum albumen and fibrin factors. The injection of water into the blood causes increased effusion, as does the presence in the circulation of certain salts and poisons. It is well known that injury to the vessels is followed by transudation of "lymph," and this injury may be brought about by blows or by chemical substances, and the latter may act from within or from without the vessels. We have, in addition, to consider the probability of another element, viz., the inherent vital properties of the tissues, and especially of the endothelial cells lining the capillaries, and possibly those coating the smaller vessels, both arterioles and venules. When a phenomenon requires chemical, physical, and so-called "vital" explanations, it must necessarily be difficult to fully understand. Two conditions, apparently very different, viz., inflammation from a wound or contusion, and Bright's disease, may be studied together to elucidate the physiology of œdema. Injury to a part is quickly followed by dilatation of the small vessels, slowing of the circulation, and increased effusion. If the injury is severe enough, there is also diapedesis of leucocytes. The broadening of the channel by enlargement of the arterioles is not enough to explain the retardation of the flow, to say nothing of the absolute "stasis" which occurs; and the mere retardation cannot account satisfactorily for the rapid and sometimes enormous effusion into the tissues. It is almost certain that some changehas been effected in the vitality of the capillary wall and perhaps in its surroundings; and this change would appear to be of the nature of "shock," either temporary or permanent. In fact, there is a "lowering of vitality," and this seems to favour passive filtration under pressure. If a small bag made of animal membrane (such as the bladder of a rat) be filled with blood clot, and introduced under the skin of a living animal, the result is this—that there is an immediate migration of leucocytes from the vessels into the clot through the *dead* animal membrane. This experiment is very instructive, for it shows, at least, that the emigration of white blood corpuscles may be explained by the lowered vitality or death of the tissues and the capillary walls. May not the inflammatory effusion or the œdema of other diseases be explained in the same way? It is not necessary, on this supposition, to attribute the changes to nerve influence, for the injury in the case cited is direct; and although the trophic function of the nerves is also destroyed by the traumatism, the result would be primarily due to immediate injury to the cells, as above stated.

In *Bright's disease* it is obvious (as Dr. Howship Dickenson has pointed out) that the œdema and other vascular phenomena cannot be explained by the hypertrophy of the smaller arteries. This would rather diminish than increase the effusion, for the obstruction would tend to limit the amount of blood to the capillaries, and would not augment the intra-capillary pressure. The fact that retinal hæmorrhages occur in the smallest arterioles would also show that some obstruction exists in the capillary, not in the arteriole system. Now, in Bright's disease the blood is always unhealthy. It is both deficient in organic constituents, and contains excess of waste products, owing to the want of proper elimination by the kidneys. The former (watery) condition favours transudation, probably partly by allowing freer osmosis, but partly also because deficient organic constituents in the circulating fluid means, of course, deficient nourishment to the capillary wall and consequent loss of vitality.

Again, the presence of urea and its predecessors in the blood would increase enormously the tendency to osmosis, and the influence of these nitrogenous metabolites would also diminish the vitality of the capillary endothelium.

We have, therefore, in two very different conditions, viz., in ordinary inflammation and in Bright's disease, circumstances which act upon the capillary wall to its detriment, and the argument is in favour of the view that œdema in either case is due to this influence rather than to any merely mechanical one.

In diseases of the heart it would seem at first that the physical states of obstruction, over-distension of the veins, &c., could easily account for the dropsy; but here there is this difficulty, that the exact cause of the obstruction is not clear. For instance, mitral regurgitation with hypertrophy of the ventricles is sometimes associated with œdema, sometimes with none, or very little. Aortic disease, both obstructive and regurgitant, may or may not be the cause of dropsy, so that one authority on heart disease has stated that whether œdema occurs must depend on some condition of the circulation *other than the disease of the heart*. This may be vasomotor paralysis; but it is equally probable that the real cause of the œdema is the imperfect state of the blood itself, and that this acts deleteriously on the delicate capillary walls.

This is rendered more probable by the fact that cardiac dropsy rarely occurs in early stages, but rather when the imperfect circulation has had time to interfere with the proper functions of the kidneys and liver, causing an accumulation in the blood of waste products. We cannot, nevertheless, ignore mechanical causes altogether; for in spite of Majendie's experiments (referred to above) there is ample proof that slowly-occuring or long-continued obstruction of either veins or capillaries *may* lead to dropsy of a part.

It has been shown that the skin of a *dead* frog will allow osmosis to go on in a manner which does not take place if the membrane is *alive*. The vitality of the cells enables them to exert a distinct influence on the amount of diffusion and on the *direction* of diffusion. This may be the case with the healthy and diseased capillaries. Fluids with organic compounds in solution may pass into the tissues in disease more readily than they will pass back again into the commencing veins. This remains to be proved, but if it is the case it may throw great light on dropsy from injury or other causes.

To sum up: There is distinct evidence that no mechanical explanation is sufficient to account for the various kinds of œdema; and there is reason to believe that some alteration in the vital properties of the endothelial capillary walls and of the surrounding tissues is an important factor in their production.

